# Religious leaders’ willingness to promote the uptake of human papillomavirus vaccine among their congregants in Mavoko Sub-County, Machakos County, Kenya

**DOI:** 10.11604/pamj.2024.48.141.43492

**Published:** 2024-07-30

**Authors:** Faith Kaaria, Felix Blair Odhiambo, Douglas Sendora Okenyoru, Lucy Murugi, Vincent Omwenga Matoke, Ruth Salima, Daniel Anyika, Gideon Ogutu, Abednego Musau

**Affiliations:** 1 Department of Community Health and Development, The Catholic University of Eastern Africa, Nairobi, Kenya,; 2 Compressive Care/TB Clinic, Partners for Health and Development in Africa (PHDA), Nairobi, Kenya,; 3 Department of Research and Learning, Population Services International, Parklands, Kenya

**Keywords:** Human papillomavirus vaccine, unwillingness, vaccine hesitancy, attitudes, religious leaders

## Abstract

**Introduction:**

cervical cancer is a significant health challenge in Kenya and sub-Saharan Africa, with high mortality linked to late presentation and low awareness. Despite its prevalence, preventive interventions like human papillomavirus (HPV) vaccination face low utilization. Religious leaders play a pivotal role in influencing health decisions, yet their attitudes toward HPV vaccination remain understudied. Therefore, this study aims to determine religious leaders' willingness to promote HPV vaccine uptake in Mavoko Sub-County, Machakos County, Kenya.

**Methods:**

a cross-sectional study involving 198 religious leaders chosen through systematic random sampling method was done in the month of December 2023 in Machakos County, Kenya. Data on socio-demographics and attitudes towards HPV vaccination were collected using structured, self-administered questionnaires. Attitudes were gauged on 11 statements. Analysis was performed using IBM SPSS 22.0, employing descriptive statistics and Chi-square tests to assess associations, with significance set at p < 0.05. Results were visually presented using tables, charts and graphs.

**Results:**

the study had a 96.6% response rate. Majority were male (51.5%), Protestant Christians (48%), and pastors (29.8%). Most participants had positive attitudes towards the HPV vaccine, with no significant socio-demographic associations found.

**Conclusion:**

the study reveals positive attitudes among religious leaders towards promoting HPV vaccine uptake among their congregants. Despite some concerns and misconceptions, the majority of participants expressed willingness to advocate for vaccination.

## Introduction

Cervical cancer is a pressing health concern in Kenya and other sub-Saharan African countries, ranking as the second most prevalent cancer among women, following breast cancer [1]. The high mortality rate associated with cervical cancer in these regions is largely due to late presentation for treatment, weaknesses in the health system, and low awareness [2,3]. Sub-Saharan Africa shoulders the highest burden of cervical cancer globally, with 21% of cases and a quarter of related deaths occurring in this region [4]. In Kenya alone, there are approximately 5,300 reported cases of cervical cancer annually, resulting in over 3,000 deaths [5]. Despite these alarming statistics, awareness and utilization of preventive measures, such as cancer screening and HPV vaccination, remain low. Studies have shown significant awareness gaps among girls aged 10-24 years, with 40% of those aged 10-14 unaware of the HPV vaccine [1,6]. By the end of 2021, only 29% of eligible girls had received at least one dose, and among them, only 44% were fully vaccinated [5]. The interplay of individual, community, and health system factors influences awareness and interest in vaccination.

Prevention strategies for cervical cancer include social and behavioral change communication, early detection through screening programs, and vaccination against the human papillomavirus (HPV), particularly serotypes 16 and 18 [7]. Despite the endorsement of HPV vaccination by the World Health Organization in 2008 and subsequent approval by Kenya's Ministry of Health [8,9], vaccine coverage remains low. In 2020, only 33% of eligible girls were vaccinated, with a mere 16% receiving the full dose. Controversies surrounding the vaccine, fueled by cultural and religious stakeholders, misinformation among parents, and inadequate awareness efforts, contribute to this suboptimal uptake [10]. Of particular concern can be the reservations expressed by religious leaders, who wield significant influence over the health decisions of their congregants. Despite these concerns, there is a lack of empirical evidence on the attitudes of religious leaders towards HPV vaccination, creating a critical research gap given their substantial impact on vaccine acceptance and the effort to achieve the 2030 cervical cancer elimination goal in Kenya. This study, conducted in Machakos County, Kenya, seeks to bridge the existing knowledge gap and determine the religious leaders´ willingness to promote the uptake of the HPV vaccine among their congregants in Mavoko Sub-County, Machakos County, Kenya. The specific objectives of the study were: i) to determine the socio-demographic factors of religious leaders associated with willingness to promote the HPV vaccine in Mavoko Sub-County, Machakos County, Kenya; ii) to assess the attitudes of religious leaders towards HPV vaccination in Mavoko Sub-County, Machakos County, Kenya

## Methods

**Study design:** this study employed a cross-sectional descriptive approach to examine religious leaders' willingness to promote the uptake of the HPV vaccine among their congregants in Mavoko Sub-County, Machakos County, Kenya. The study design aimed to determine the religious leaders' willingness and attitudes toward the promotion of HPV vaccine uptake among their congregants at that particular time.

**Setting:** the study was conducted in Mavoko Sub-County, situated within Machakos County, Kenya. Mavoko Sub-County was purposively selected as the study setting due to its diverse population and concentration of religious institutions, which provided ample opportunities to engage with religious leaders on attitudes towards and promotion of HPV vaccination. The area's demographic and socio-cultural characteristics made it an ideal location to explore religious leaders´ willingness to promote the uptake of HPV vaccines among their congregants.

**Participants:** the study population comprised religious leaders, including priests, catechists, deacons, pastors, evangelists, bishops, imams, sheikhs, and others. Participants were required to meet specific inclusion criteria, including serving as religious leaders for at least three months, being aged 18 and above, providing voluntary written consent, and being available for an interview. Individuals unwilling to provide written consent or unavailable for an interview during the study period were excluded.

**Enrollment of participants:** participants were selected using systematic random sampling from a list of 500 religious leaders given by the Movoko Sub-County religious leaders association. Using a sample of 205, a skip interval of 2 was used in the selection process after identification of a random start point leaving those who are not eligible and moving to the next person meeting the inclusion criteria. This was continued until the required sample size was achieved.

**Inclusion and exclusion criteria:** the inclusion criteria required participants to have been serving as religious leaders for at least three months, to be aged 18 and above, and to be available for data collection during the study period. Exclusion criteria included individuals who were unwilling to participate or were seriously ill.

**Variables:** the main outcome variable of interest in this study was the religious leaders´ willingness to promote the uptake of the HPV vaccine among their congregants. This variable was assessed using a structured questionnaire administered to participants across various religious institutions in Mavoko Sub-County, Machakos County, Kenya. The questionnaire was designed to capture attitudes towards and readiness to advocate for HPV vaccination among congregants. Specifically, participants were asked about their agreement or disagreement with statements related to HPV vaccination, which were scored to categorize attitudes as positive or negative. Respondents scoring above 1.5 were categorized as having a positive attitude, indicating a willingness to promote the vaccine, while those scoring below 1.5 were categorized as having a negative attitude, reflecting unwillingness. The tool was designed to capture both socio-demographic factors and attitudes through validated questions derived from a literature review and pre-tested for reliability and validity in a similar peri-urban setting. This approach aimed to ensure consistency in data collection and minimize bias, thereby providing robust insights into the factors influencing religious leaders' engagement with HPV vaccination promotion

**Data sources and data collection:** data were collected through a well-structured questionnaire that was self-administered by trained research assistants at the church in December 2023. The questionnaire was organized into two sections: Section A focused on socio-demographic factors such as age, religion, marital status, number of children, level of education, occupation, monthly income, and residence. Section B assessed attitudes using 11 statements with response options categorized as “Agree,” “Neutral/Undecided,” or “Disagree.” Average scores were calculated, and respondents scoring below 1.5 were categorized as having negative attitudes, while those scoring above 1.5 were categorized as having positive attitudes.

**Bias:** the study was susceptible to information bias due to potential non-response. To mitigate this, the questionnaires underwent pre-testing for validity and reliability, which were conducted with a well-structured questionnaire self-administered by trained research assistants at the church in October 2023. The pretest was conducted in Kajiado North Sub-County, a peri-urban environment similar to the study area, to ensure consistency and reduce bias from question inconsistencies. Additionally, systematic random sampling was employed to select participants, further minimizing bias. The statements were generated based on a thorough literature review.

**Study size:** the sample size was determined using Fishers's formula [11], resulting in a total of 205 participants from various religious domains.


$$n=\frac{Z^{2}P\left(1-P\right)}{E^{2}}$$

The sample size calculation was based on a 95% confidence level with a normal deviation (Z) of 1.96. Willingness of religious leaders to promote the uptake of the HPV vaccine (35%), (P = 0.35), with the proportion of unwillingness of religious leaders to promote the uptake of the HPV serving as a measure of variability (Q = 1-P). The allowable error range (E) was set at 5% (0.05). Applying these values to the formula, the initial estimated sample size was 349 participants. To accommodate for potential non-response and incomplete surveys, the sample size was increased by 10%, resulting in a final sample size of 384 participants. For a population below 10,000 subjects, Cochran's formula [12], was utilized to adjust the size sampled. The Mavoko Pastor´s Association estimated that there are approximately 500 religious leaders in the area. Using for sample size reduction specifically the finite population correction:


(nf)=n/(1+n/N)

Where; n = 349 and N = 500, the standardized sample size was calculated to be 205 participants. Here, n = the desired sample size when the population exceeds 10,000; (nf) = the adjusted sample size when the population is below 10,000; and N = the estimated number of religious leaders in the selected study area, Mavoko Sub-County.

**Quantitative variables:** quantitative variables included participants' demographic characteristics, such as age and duration of service as a religious leader, as well as scores indicating attitudes towards the HPV vaccine. Frequencies were used to summarize the demographic characteristics. Attitudes were scored based on these responses, with scores ranging from 11 to 33. A score above 1.5 was categorized as having a positive attitude, indicating a willingness to promote the vaccine, while those scoring below 1.5 were categorized as having a negative attitude, reflecting unwillingness.

**Statistical methods:** data cleaning and analysis were performed using IBM SPSS 22.0. Descriptive statistics, including frequencies and proportions, were used to summarize the data. Inferential analysis, including the Chi-square test, was employed to assess associations between variables, with a significance level set at p < 0.05.

**Ethical consideration:** ethical approval was obtained from the Scientific Ethics and Review Committee (SERC) of the University of Nairobi/Kenyatta National Hospital (UoN/KNH) with approval number UP66/01/2023. Additionally, written authorization was obtained from the Machakos County Department of Health and the Mavoko Sub-County Commissioner (part of the national administration). Local administration (Chiefs and Sub-chiefs) in the ward were informed about the study before data collection commenced. Written informed consent was obtained from all participants, and confidentiality was ensured throughout the study process.

## Results

**Distribution of socio-demographic factors:** this investigation aimed to show how respondents' social-demographic characteristics were distributed. The overall response rate was 96.6%. Overall, results indicated that the majority of participants were male: 102 (51.5%) and 96 (48.5%) were female. In terms of marital status, 134 (67.7%) were married, followed by single or unmarried 34 (17.2%) participants. The majority of the participants identified as Protestant Christians, 95 (48%), followed by Roman Catholics, 51 (25.8%). In terms of the role held in the institution, pastors had a plurality of 59 (29.8%), followed by elders with 31 (15.5%) participants. In regards to education level, those with a degree in theology were the majority (66, 33.3%), followed by those with a diploma, 58 (29.3%) participants. The results are shown in Table 1.

**Table 1:** participants’ social-demographic factors (n=198)

Variable	Categories	Frequency	Proportion
**Gender**	Male	102	51.5%
	Female	96	48.5%
**Marital status**	Married	134	67.7%
	Single/not married	34	17.2%
	Separated/widowed/divorced	21	10.6%
	Cohabiting	9	4.5%
**Religion**	Protestant	95	48.0%
	Roman catholic	51	25.8%
	Seventh Day Adventist	32	16.2%
	Muslim	20	10.1%
**Role in religious institution**	Bishop	15	7.6%
	Brother/sister	19	9.6%
	Catechist	13	6.6%
	Deacon	14	7.1%
	Elder	31	15.5%
	Imam	11	5.6%
	Pastor	59	29.8%
	Priest	16	8.1%
	Reverend	20	10.1%
**Level of education**	Degree in theology	66	33.3%
	Diploma	58	29.3%
	Learning by experience	41	20.7%
	Certificate	26	13.1%
	Other training	7	3.5%

**Association between social-demographic factors and willingness to promote the human papillomavirus vaccine:** the study aimed to assess whether socio-demographic characteristics and willingness to promote the HPV vaccine. Notably, the results suggest that in terms of sex, female participants had the highest proportion of willingness to promote the HPV vaccine, 28(14.2%), followed by males, 31(15.7%), with no significant association between gender and desire to promote the HPV vaccine (P = 0.457). According to religious affiliation, Christians had a higher proportion of participants who were willing to promote the HPV vaccine 30 (15.3%), followed by Roman Catholics 12 (6.1%), indicating no statistically significant association between religion and willingness to promote the HPV vaccine (P = 0.700). In regards to education level, those who attended university or college had a higher proportion of willingness to promote the HPV vaccine, 34 (17.3%), followed by those with tertiary or vocational education, 13 (6.6%), indicating no statistically significant association between education level and willingness to promote the HPV vaccine (P = 0.124). In terms of institutional positions, pastors had the largest proportion of willingness to promote the HPV vaccine, 14 (7.1%), followed by reverend, 8 (4.1%), demonstrating that there is no statistically significant association between institutional role and readiness to promote the HPV vaccine (P = 0.801). According to the type of training, those with a degree in theology were the most willing to promote the HPV vaccine, 19 (9.7%), followed by those with a diploma, 18 (9.2%), with no statistically significant association between type of training and willingness to promote the HPV vaccine (P = 0.738). The results are presented in Table 2.

**Table 2:** association between social-demographic factors and willingness to promote the human papillomavirus vaccine (n=198)

Variable/categories		Willingness to promote the human papillomavirus (HPV) vaccine		Chi-square degree of freedom p-value
		No; (n=139, %= 70.2)	Yes; (n=59, %= 29.8)	
**Sex**	Female	65(32.7%)	31(15.7%)	X^2^=554; DF=1; P=0.457
	Male	74(37.3%)	28(14.2%)	
**Religion**	Muslim	14(7.1%)	6(3.1%)	X^2^=1.423; DF=3; P=0.700
	Protestant/Christian	65(32.7%)	30(15.3%)	
	Roman catholic	39(19.6%)	12(6.1%)	
	Seventh day	21(10.6%)	11(5.6%)	
**Level of education**	Non-formal	0(0.0%)	1(0.5%)	X^2^=7.228; DF=4; P=0.124
	Primary	0(0.0%)	1(0.5%)	
	Secondary	14(7.1%)	10(5.1%)	
	Tertiary/vocational	41(20.6%)	13(6.6%)	
	College/university	84(42.3%)	34(17.3%)	
**Role in the institution**	Bishop	9(4.5%)	6(3.1%)	X^2^=4.583; DF=8; P=0.801
	Brother/sister	14(7.1%)	5(2.5%)	
	Catechist	9(4.5%)	4(2.0%)	
	Deacon	9(4.5%)	5(2.5%)	
	Elder	24(12.1%)	7(3.6%)	
	Imam	7(3.5%)	4(2.0%)	
	Pastor	45(22.7%)	14(7.1%)	
	Priest	10(5.0%)	6(3.1%)	
	Reverend	12(6.0%)	8(4.1%)	
**Type of training**	Certificate	16(8.1%)	10(5.1%)	X^2^=1.985; DF=4; P=0.738
	Degree in theology	47(23.7%)	19(9.7%)	
	Diploma	40(20.1%)	18(9.2%)	
	Learning by experience	30(15.1%)	11(5.6%)	
	Other training	6(3%)	1(0.5%)	

**Attitudes towards the human papillomavirus vaccine:** attitudes towards the HPV vaccine were further analyzed by assessing participants' responses to specific statements. The results on attitudes toward cervical cancer and the HPV vaccine reveal that the majority of respondents hold positive views towards the vaccine and reject misconceptions about cervical cancer. A significant proportion (93.4%) disagreed with the statement that cervical cancer occurs due to immoral behavior, averaging a score of 2.05. Similarly, 97.5% disagreed with the notion that those who get cervical cancer deserve it, with an average score of 2.02. Most participants (88.9%) rejected the idea that it is unethical to administer the HPV vaccine to healthy girls, scoring 2.05. A strong majority (93.4%) agreed that the HPV vaccine does not encourage promiscuity, with a low score of 1.07, indicating negative attitudes towards the misconception. Additionally, 89.4% disagreed that vaccinating girls against HPV would decrease condom use and increase STIs, scoring 1.12. Concerns about the vaccine causing health problems were less common, with 69.2% disagreeing and an average score of 2.61. The belief that the HPV vaccine would lead to more HIV cases was also rejected by 97% of respondents, averaging 2.03. Obtaining the HPV vaccine was not seen as difficult, with 72.7% disagreeing, resulting in a score of 2.72. The vaccine was not considered burdensome by 90.4% of participants, scoring 2.90. Most respondents (88.4%) disagreed that the HPV vaccine is not safe and effective, with an average score of 1.14. Finally, 96% rejected the statement that the HPV vaccine could not have a positive impact on a girl´s life, scoring the lowest average of 1.05. Overall, the average score across all statements was 1.89, indicating a generally positive attitude towards the HPV vaccine. Results are as shown in Table 3.

**Table 3:** individual responses to the scale on attitudes towards the human papillomavirus vaccine statements (n=198)

Statement	Agree	Neutral/undecided	Disagree	Average score	Level attitude
Cancer of the cervix occurs due to immoral behavior	2(1%)	185(93.4%)	11(5.6%)	2.05	Positive
People who get cancer of the cervix get what they deserve	1(0.5%)	193(97.5%)	4(2%)	2.02	Positive
It is unethical to give medicines such as the human papillomavirus vaccine (HPV) vaccine to healthy girls	6 (3%)	176 (88.9%)	16 (8.1%)	2.05	Positive
The HPV vaccine does not encourage promiscuity	185(93.4%)	13(6.6%)	0(0%)	1.07	Negative
Vaccinating girls against HPV will lead to a decrease in condom use and an increase in sexually transmitted infections (STIs)	177(89.4%)	21(10.6%)	0(0%)	1.12	Negative
I am very worried that HPV vaccine can lead to health problems	17(8.6%)	44(22.2%)	137(69.2%)	2.61	Positive
Human papillomavirus vaccine vaccine will lead to an increase in HIV cases	0(0%)	192(97%)	6(3%)	2.03	Positive
It is difficult and tedious to obtain the HPV vaccine	1(0.5%)	53(26.8%)	144(72.7%)	2.72	Positive
It is burdensome to take the HPV vaccine	1(0.5%)	18(9.1%)	179(90.4%)	2.90	Positive
The HPV vaccine is not completely safe and effective in preventing cancer of the cervix	175(88.4%)	18(9.1%)	5(2.5%)	1.14	Negative
Taking the HPV vaccine could not have a positive impact on a girl’s life	190(96%)	7(3.5%)	1(0.5%)	1.05	Negative
Overall score				1.89	Positive

## Discussion

**Socio-demographic characteristics:** regarding sex, the study revealed that the majority of religious female leaders expressed a willingness to promote HPV vaccine uptake. However, the observation revealed an approximately equal split between female and male leaders, implying that both male and female religious leaders may have comparable perspectives shaped by their religious teachings or interpretations, which may impact their position on HPV vaccination. These findings call into question widespread preconceptions that attribute vaccine-related attitudes to a specific gender. Both boys and females showed equivalent degrees of refusal, showing a shared complexity in their perspectives on the HPV vaccine [13].

In terms of religion, the study showed that Protestant Christians had the highest willingness, followed by Roman Catholics. This suggests that the observed differences in willingness across religious groups could be due to the diversity and internal heterogeneity within religious groups regarding health beliefs and practices. These findings challenge any simplistic assumptions regarding the impact of religious beliefs on attitudes toward vaccination. It highlights the complexity of vaccine-related decision-making, which is influenced by a myriad of factors beyond religious affiliation [14,15]. According to educational levels, the majority of participants who exhibited the highest willingness had a college or university level of education. This suggests that the observed differences in willingness across educational levels could be attributed to disparities in access to information and varying levels of health literacy among different educational strata within the studied population. These findings underscore the complexity of the relationship between educational background and vaccine-related attitudes. They challenge any simplistic assumptions that higher levels of education universally correlate with positive attitudes toward vaccination [16]. However, this finding contradicts a study conducted in Puerto Rico, which suggests that HPV knowledge and vaccination awareness are associated with the level of education [17].

Regarding their role in the institution, pastors exhibited the highest willingness to promote the HPV vaccine. This suggests that the observed differences in willingness across roles within the institution could arise from the nuanced dynamics of leadership and decision-making within religious contexts. These findings highlight the intricate nature of the relationship between religious roles and attitudes related to vaccines. They challenge any simplistic assumptions about the impact of specific roles within religious institutions on vaccination advocacy [18]. Lastly, in terms of the type of training, individuals with a degree in theology demonstrated the highest willingness. This suggests that the observed differences in willingness across training categories could be due to the educational preparation and background of the religious leaders. These findings highlight the complexity of the relationship between the specific type of training received and attitudes related to vaccines. They challenge assumptions that certain types of formal training universally correlate with more positive attitudes toward vaccination [14].

**Attitude:** analyzing attitudes towards the HPV vaccine, the results revealed that the majority of respondents were neutral or undecided about the belief that cervical cancer occurs due to immoral behavior. This could be attributed to the influence of deeply ingrained cultural and societal beliefs. This finding is consistent with a study conducted among adult US women [19] and emphasizes a prevailing uncertainty in this cohort of religious leaders. This uncertainty could be rooted in diverse religious teachings and ethical perspectives regarding health and disease [18]. Similarly, a significant portion of individuals expressed neutrality or were undecided regarding the idea that those with cervical cancer deserve their condition. This could be attributed to the coexistence of compassionate religious teachings with stigmatizing cultural beliefs. This discovery suggests a possible gap between attributions of personal responsibility and the comprehension of disease causation within this religious group. It also indicates widespread uncertainty or a lack of consensus among respondents regarding the deservingness of cervical cancer. The study's findings are not in line with the assertion made by Bouchez and colleagues [20] in their study, which suggests that the population has not lost faith in public health.

Furthermore, a significant proportion were neutral or undecided on the ethics of administering the HPV vaccine to healthy girls. This could be due to a lack of comprehensive education or information about the vaccine's safety and benefits. This suggests a need for nuanced discussions within religious communities about the ethical considerations surrounding preventive health measures, such as vaccinations. The study aligns with Wyndham-West´s study [21], indicating a considerable level of uncertainty or concern among respondents regarding the ethical aspects of vaccinating healthy individuals.

**Limitations:** the study exclusively focused on religious leaders, suggesting that involving diverse community members could have provided more comprehensive findings. The absence of an association between socio-demographic factors and willingness to promote HPV vaccination among religious leaders may indeed be influenced by the study's narrow focus.

## Conclusion

Underscores the positive attitudes of religious leaders towards promoting HPV vaccination among their congregants. Despite some initial concerns and varying demographic characteristics, such as gender, religion, education, and role within religious institutions, the majority of participants displayed a willingness to advocate for HPV vaccination.

### What is known about this topic

- *Religious leaders in some dominations and communities may express hesitancy or unwillingness to promote the HPV vaccine among their congregants due to concerns about perceived moral implications associated with the vaccine;*
- *Cultural and religious beliefs may contribute to skepticism or resistance towards vaccination programs, including HPV vaccination, among certain religious groups;*
- *Lack of awareness about the efficacy and safety of the HPV vaccine, coupled with misconceptions about its purpose and potential side effects, can further deter religious leaders from actively promoting its uptake among their followers.*

### What this study adds

- *This study highlights that willingness to promote the HPV vaccine is not significantly associated with socio-demographic factors such as sex, religion, education, institutional role, or type of training;*
- *This study highlights a clear association between positive attitudes towards the HPV vaccine and increased willingness to promote its uptake, emphasizing the importance of addressing misconceptions to enhance vaccine acceptance and public health outcomes;*
- *Highlights the potential role of religious leaders in improving HPV vaccine awareness and uptake among communities in Machakos County, Kenya.*
